# Clinical, radiological, and surgical outcomes of 431 patients with adrenal incidentalomas: retrospective study of a 10-year single-center experience

**DOI:** 10.55730/1300-0144.5802

**Published:** 2024-03-11

**Authors:** Narin NASIROĞLU İMGA, Yılmaz ASLAN, Merve ÇATAK, İbrahim Can AYKANAT, Altuğ TUNCEL, Dilek BERKER

**Affiliations:** 1 Department of Endocrinology and Metabolism, University of Health Sciences, Ankara City Hospital, Ankara, Turkiye; 2 Department of Urology, Üsküdar University Faculty of Medicine, Medicana Ataköy Hospital, İstanbul, Turkiye; 3 Department of Endocrinology and Metabolism, Tokat Gaziosmanpaşa University, Faculty of Medicine, Tokat, Turkiye; 4 Department of Urology, Koç University Faculty of Medicine, İstanbul, Turkiye; 5 Department of Urology, University of Health Sciences, Ankara City Hospital, Ankara, Turkiye; 6 Department of Endocrinology and Metabolism, University of Health Sciences, Retired Lecturer, Ankara, Turkiye

**Keywords:** Adrenal incidentaloma, nonfunctioning, functioning

## Abstract

**Background/aim:**

The incidence of adrenal tumors is increasing due to the widespread utilization of radiographic imaging techniques. Factors such as tumor size, radiological characteristics, and functionality of adrenal adenomas play crucial roles in diagnosis and subsequent management. In this retrospective study, we investigated the clinical, radiological, and surgical features of patients with adrenal incidentalomas (AIs) and evaluated their follow-up results.

**Materials and methods:**

We analyzed data from 431 patients diagnosed with AIs (130 males, 301 females) who underwent adrenal hormone evaluation at our center. We compared nonfunctioning and functioning AIs in terms of radiological features. We also compared baseline and follow-up characteristics in nonfunctioning AIs.

**Results:**

The mean age of the patients was 55.4 ± 11.5 years, with a mean tumor size of 25.9 ± 14.3 mm. Mean follow-up duration was 3.17 ± 2.07 years. Adenoma localization revealed 165 (38.3%) right-sided, 185 (42.9%) left-sided, and 81 (18.8%) bilateral cases. Most patients (76.6%) had nonfunctioning AIs. During follow-up, nonfunctioning AIs exhibited increased fasting blood glucose, fasting insulin and HOMA-IR values (p = 0.002, <0.001 and 0.004, respectively). Among the functioning AIs cases (23.4%), autonomous cortisol secretion, Cushing’s syndrome, pheochromocytoma, and primary aldosteronism were observed in 10.4%, 5.1%, 3.9%, and 3.9% of cases, respectively. Receiver operating characteristic curve analysis determined an adrenal adenoma size of 26.5 mm as the optimal cut-off for distinguishing between functioning and nonfunctioning AIs, with a sensitivity and specificity of 61.4% and 70.0%, respectively.

**Conclusion:**

Although the majority of AIs are nonfunctioning, the prevalence of functioning adrenal adenomas is not rare. Our findings suggest that adenoma size emerges as a valuable predictor for early detection of functioning adenomas. In addition, smaller masses appear to carry a lower risk of malignancy.

## 1. Introduction

Adrenal incidentalomas (AIs) are often identified incidentally during crosssectional abdominal imaging performed for reasons unrelated to adrenal gland disorders [1]. Studies conducted over the years have shown that the frequency of AIs has increased. This increase, with a nearly 10-fold increase in standardized incidence rates, has been attributed to the widespread utilization of abdominal magnetic resonance imaging (MRI) and computed tomography (CT) scans [2]. Notably, analyses of CT scans have reported an approximate 5% prevalence of AIs [3]. Studies of autopsy series have reported the prevalence of adrenal masses to be between 1.0% and 8.7% [4]. Both radiological and autopsy data consistently demonstrate an age-related increase in the prevalence of AI, reaching its peak between the 5th and 7th decades of life and being less prevalent in individuals under 30 years of age [5].

It is recommended that the evaluation of adrenal adenomas include concurrent assessments for both hormone excess and malignancy risk [5,6]. Tumor size, radiological features, and hormonal status of adrenal adenoma are crucial factors in the management of disease at the time of diagnosis and during follow-up. While the majority of AIs are nonfunctioning and benign, usually not requiring any therapeutic intervention, a subset demonstrates secretory activity, termed functioning adenomas. A diagnostic evaluation for adrenal hormone excess includes measuring plasma or urinary metanephrines, conducting 1-mg overnight dexamethasone suppression test, and, in cases with concurrent hypertension or unexplained hypokalemia, evaluating aldosterone and plasma renin activity.

Recent studies between healthy groups and nonfunctioning AI patients have not definitively established a cause-and-effect relationship between diabetes, hypertension, and insulin resistance. Such potential associations may be due to insidious chronic exposure to low cortisol hypersecretion [7].

In this retrospective study, we investigated the clinical, radiological, and surgical characteristics, as well as the follow-up results, of patients diagnosed with AIs. Furthermore, we compared nonfunctioning and functioning adrenal adenomas in terms of size and radiological features. Additionally, we aimed to identify a critical tumor size threshold that predicts functioning AIs.

## 2. Materials and methods

This retrospective analysis was performed on medical records of patients diagnosed with AIs. Ethical approval for the study was obtained from the local ethics committee (approval number: ANEAH–E–17-1402). Patients aged 18 years and older, who were diagnosed with adrenal incidentaloma and underwent a complete hormonal work-up, were retrospectively evaluated. Patients with insufficient data regarding radiological or hormonal evaluations were excluded from the analysis. Based on this approach, the data from 672 AIs subjects who attended our clinic between January 2009 and January 2019 were evaluated. Ultimately, the data from 431 patients who had been followed up consistently were included in the study. Information on medical history, physical examination, laboratory tests, and radiological parameters were recorded. Anthropometric measurements, including height (cm), weight (kg), and blood pressure (mmHg), were obtained. Body mass index (BMI) was calculated as kg/m^2^. The diagnosis of functioning adrenal incidentalomas were made based on the clinical history and hormonal workup results in accordance with AI guidelines [5,6,8,9]. Radiological findings of adrenal adenoma were reviewed, and the largest size of the tumor was measured. Additionally, HU and tumor washout percentage were determined.

Routine biochemical evaluation after an overnight fasting encompassed parameters such as fasting blood glucose (FBG), fasting insulin, total cholesterol, low-density lipoprotein cholesterol (LDL-C), high-density lipoprotein cholesterol (HDL-C), and triglyceride levels. Homeostasis model assessment of insulin resistance (HOMA-IR) was calculated using FBG and fasting insulin levels, with the formula HOMA-IR = (FBG × fasting insulin). Adrenal hormone evaluations included measurements of adrenocorticotropin hormone (ACTH), dehydroepiandrosterone sulfate (DHEA-S), 24-h urinary fractionated metanephrines, and 1 mg overnight dexamethasone suppression test (DST). Plasma renin activity and aldosterone levels were evaluated for primary hyperaldosteronism in hypertensive patients. Serum ACTH was measured using the immunochemiluminometric assay method. The level of 24-h urine free cortisol was measured with an LC-MS method. Midnight salivary cortisol, plasma cortisol and 17-hydroxiprogesterone levels were determined using the Cobas e601 electrochemiluminescence immunoassay system (Roche Diagnostics, Mannheim, Germany). Autonomous cortisol secretion was excluded by the suppression of the morning cortisol levels (≤1.8 μg/dL) after overnight administration of 1 mg dexamethasone. A diagnosis of pheochromocytoma was made if 24-h urinary fractionated metanephrine levels were above the normal range. Assessment for primary hyperaldosteronism was conducted based on plasma aldosterone, renin, and potassium levels. The plasma aldosterone concentrations >15 ng/dL, plasma renin activity <1.0 ng/mL/h and an aldosterone-renin ratio (ARR) >20 ng/mL/h were found significant for the diagnosis of primary hyperaldosteronism. Confirmation of primary aldosteronism was established based on either the saline infusion test or the captopril challenge test.

### 2.1. Statistical analysis

Statistical analysis was performed using the SPSS version 15 (SPSS Inc., Chicago, IL, USA). Categorical variables are presented as numbers or percentages, while normally distributed continuous variables are expressed as mean ± standard deviation. Continuous variables between groups were evaluated using the independent t-test for normally distributed variables and the Mann-Whitney U test for nonnormally distributed variables. Categorical variables were compared using the chi-square test and Fisher’s exact test. Histogram and the Kolmogorov-Smirnov tests were performed to determine the distribution of patients. Additionally, a receiver operating characteristic (ROC) curve analysis was performed to determine the optimal tumor size cut-off for functioning AIs that provided the best diagnostic accuracy in predicting functioning adenomas. A p value of less than 0.05 was considered statistically significant.

## 3. Results

The demographic and clinical features of all patients are summarized in Table 1. The mean age of the patients was 55.4 ± 11.5 years. Most of the patients were female (69.8%). Hypertension was found to be the most common comorbidity, followed by diabetes mellitus. Prevalence rates for hypertension, diabetes mellitus, prediabetes, hyperlipidemia, and coronary artery disease were 23.9%, 7.2%, 3.7%, 6.3%, and 1.4%, respectively. The mean tumor diameter for AIs was 25.9 ± 14.3 mm, with 87.7% measuring less than 40 mm (n = 378). Localization analysis of adenomas revealed that 165 (38.3%) were right-sided, 185 (42.9%) were left-sided, and 81 (18.8%) were bilateral. Examination of tumor density on CT scans indicated that 294 (79.2%) of the lesions had a Hounsfield unit (HU) of 10 or less. This finding shows that the majority of patients have benign adrenal masses. Specifically, shape irregularity, heterogeneous appearances, necrosis, and invasion were observed in 2.3%, 14.8%, 1.4%, and 0.7% of cases, respectively.

**Figure 1:**
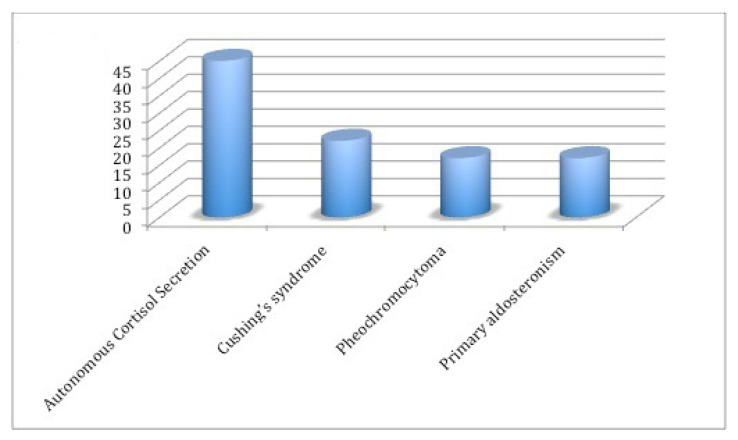
Functioning adrenal incidentalomas patient distribution on the basis of adrenal hormone excess.

**Figure 2:**
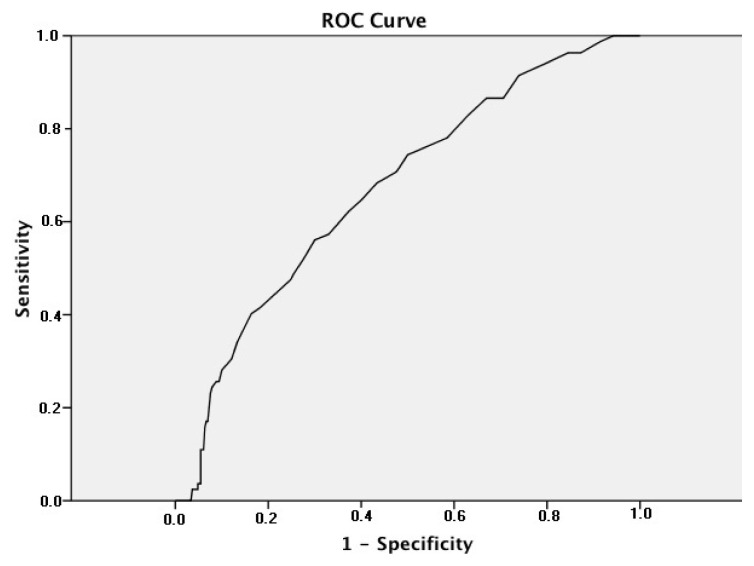
ROC curve with the functioning adrenal incidentalomas for the detection of predicting functioning adenoma tumor size.

Patients were categorized into nonfunctioning and functioning adenoma groups based on hormonal work-up assessments. Table 2 presents a comparison analysis between nonfunctioning AIs and functioning AIs groups. Of the total patients, 330 patients (76.6%) exhibited nonfunctioning AIs, while 101 (23.4%) had functioning AIs. Notably, a significant majority of functioning AIs were female (p = 0.009). In terms of radiological characteristics, the mean baseline and follow-up tumor diameter was larger in patients with functioning AIs compared to those with nonfunctioning AIs (29.9 ± 14.1 vs. 23.3 ± 13.0, p < 0.001). Increase in tumor size in functioning adenomas was significantly higher than in nonfunctioning adenomas (−0.50 ± 5.8 vs. 1.8 ± 5.4 mm, p = 0.004). Moreover, the functioning group exhibited a greater variation in mass size during follow-up. This suggests that the probability of hormone secretion increases in larger adrenal masses. A noncontrast CT density HU ≤10 indicates a potentially benign feature of AIs. When comparing this feature between nonfunctioning and functioning groups, it was more prevalent among nonfunctioning AIs (83.3% vs. 52.5%, p < 0.001). As patients with functioning AIs typically undergo surgery promptly after diagnosis, their follow-up durations were relatively short. Patients diagnosed with nonfunctioning AIs were usually followed. Our nonfunctioning AIs patients were regularly monitored for over 3 years (3.17 ± 2.07 years). The baseline and follow-up characteristics of patients with nonfunctioning AIs are detailed in Table 3. The baseline and follow-up mean tumor sizes did not show statistically significant differences among the right-sided, left-sided, and bilateral AIs groups. Throughout the follow-up period, most laboratory parameters remained stable, with notable elevations observed in FBG, fasting insulin, and HOMA-IR levels (p = 0.002, <0.001, and 0.004, respectively). HDL-C levels exhibited an increasing trend in the lipid profile during follow-up, while no significant changes were observed in total cholesterol, triglyceride, and LDL-C levels. As shown in Figure 1, frequency of functioning AIs was 23.4% among all AIs, with distributions of adrenal hormone excess as follows: 10.4% (n = 45) for autonomous cortisol secretion, 5.1% (n = 22) for Cushing’s syndrome, 3.9% (n = 17) for pheochromocytoma, and 3.9% (n = 17) for primary aldosteronism. Among the functioning adrenal adenoma group, 96 out of 101 patients underwent adrenalectomy operation. The remaining five patients who did not undergo an operation had primary hyperaldosteronism and received medical treatment. Four of our patients with adenoma size greater than 6 cm were operated on. Among them, three were diagnosed with adrenocortical carcinoma, while one patient was found to have primary adrenal B-cell lymphoma. During the follow-up period, 12 (3.6%) of the hormonally inactive AIs were subsequently found to exhibit hormonal activity. As shown in Figure 2, receiver operating characteristic curve (ROC) analysis revealed that the optimal tumor size cut-off for distinguishing between functioning and nonfunctioning AIs is 26.5 mm, with a sensitivity of 61.4% and specificity of 70.0%. The area under the curve (AUC) was statistically significant [AUC = 0.694 (95% CI: 0.635–0.753), p < 0.001].

## 4. Discussion

The management of AIs is a common issue for clinicians, especially considering their rising incidence due to the widespread use of imaging methods in recent years. In addition, due to the overuse of thorax CT during the COVID-19 pandemic, a higher prevalence of incidentally detected adrenal masses was observed[10]. Notably, the majority of AIs are nonfunctioning and benign lesions, posing no clinical implications for patients [11].

This study has shown several features of AIS during the follow-up period. Primarily, our analysis revealed that the majority of patients in our cohort had nonfunctioning AIs (76.6%). The prevalence range for nonfunctioning Ais, as stated in a recently published guideline on adrenal incidentaloma management, falls between 40% and 70% [12]. Consistent with our findings, a recent study reported a 72.6% frequency of nonfunctioning AIs [13]. Studies have reported different percentages for the localization of adenomas within the adrenal gland [14]. Although many studies have reported a similar distribution of adrenal tumors between the right and left adrenal glands [15], our investigation revealed a higher prevalence of adenomas originating from the left adrenal gland compared to the right adrenal gland (42.9% vs. 38.3%). Consistent with our findings, several studies have also showed a higher prevalence of left-sided adrenal tumors identified through imaging [16,17]. We found that the percentage of bilateral AIs was 18.8%. There were no changes in baseline and follow-up tumor sizes among the right-sided, left-sided, and bilateral groups. If there is evidence of hyperandrogenemia with bilateral adrenal masses, congenital adrenal hyperplasia, adrenal insufficiency, and malignancy should be considered.

Hormone excess is a major concern in the management of AIs. It is recommended that each patient with an adrenal incidentaloma undergoes a careful assessment, including clinical examination for symptoms and signs of adrenal hormone excess. Several studies have reported that the female sex is a predisposing risk factor for functioning AIs [18]. In our study, the percentage of females was higher among functioning AIs compared to the nonfunctioning group. The prevalence of autonomous cortisol secretion among AIs was found to be between 20% and 50%. The data from our study showed that autonomous cortisol secretion was the most common function, consistent with our previous study [19]. Among all AIs in our cohort, autonomous cortisol secretion was the most common functional mass, with a frequency of 10.4%. The observed lower frequency may stem from expanded criteria and delayed appearance of clinical findings for autonomous cortisol secretion (formerly known as subclinical Cushing’s syndrome) over the years. In a multicenter retrospective study, the autonomous cortisol secretion of 5.7% was lower than in our patients [20].

The size of adrenal tumors is crucial for evaluating malignancy risk. In patients who underwent CT scans, a size of 4 cm with HU greater than 20 was identified as the primary indicator of malignant adrenal lesions [12]. In our cohort of AIs patients, the mean tumor diameter size was 25.9 ± 14.3 mm, slightly less than the reported mean diameter of 30 mm for AIs cases detected via CT scans [15]. Prior studies have indicated that most nonfunctioning adrenal lesions exhibited no significant change in size over the follow-up period [21]. In our study of patients with functional AIs, the baseline adenoma size was larger than in patients with nonfunctioning AIs. As tumor size increased, the mass tended to be functional. Specifically, 62.9% of adrenal masses measuring 4–6 cm were functional, while the risk of malignancy increased to 33.3% in masses larger than 6 cm. Therefore, surgery may be a crucial option for patients with a 4–6 cm adrenal mass. The tumor size of AIs may serve as a potentially value predictor for estimating excess hormone production. The frequency of adrenocortical carcinoma was found to be higher in adenomas with a diameter of larger than 4 cm, with adrenocortical hormone excess, and at younger ages [22]. Another important finding of our study is that tumor size was larger in functioning AIs than in nonfunctioning AIs in both the baseline and follow-up periods. There was also a significant difference in tumor mass size variation during follow-up between the groups. During the follow-up period, the enlargement of tumor size in functioning adenomas was significantly higher than in nonfunctioning adenomas. Therefore, if functioning AIs cannot be operated on, they require careful monitoring. Additionally, >10 HU with tumor contrast washout <50% is more frequently observed in functioning AIS. In the literature, alterations in the tumor size of AIs have been reported [23]. There is a low risk of tumor size enlargement for nonfunctioning AIs, and there are no established criteria for predicting tumor size during the follow-up period. In our opinion, the risk of malignancy and tumor enlargement, as well as baseline high HU levels, are considered the two most important factors for the management of AIs. We found 26.5 mm to be the optimal cut-off for discriminating between functioning and nonfunctioning AIs. A study reported that a size of adenoma greater than 2.4 cm was associated with the risk of developing subclinical hypercortisolism [24]. During the follow-up period, we observed an increase in fasting blood glucose, fasting insulin, and HOMA-IR in the nonfunctioning adenoma group. Similar results were obtained in previous studies [25,26]. Additionally, a recent study indicated that nonfunctioning AIs had a 2-fold higher risk of insulin resistance compared to the control group [27]. Insulin resistance may increase in nonfunctioning AIs patients over the years, even if it was not present initially.

Our study has some limitations. First, as a retrospective study, there were limitations in accessing specific data (smoking history, menopause status, etc.). The second limitation is that radiological imaging scans were not consistently conducted by the same radiologist throughout the follow-up period.

In summary, our findings suggest that the majority of adrenal masses are nonfunctioning and benign lesions; therefore, they do not require surgery. However, if there are signs and symptoms of cortisol excess, uncontrolled hypertension and hypokalemia, monitoring and hormonal evaluations should be performed during the follow-up period. Further laboratory investigations are necessary to determine the natural course of AIs during follow-up. In conclusion, our study data indicates that adenoma size may serve as a valuable marker for predicting the early detection of functioning adenomas. Although the vast majority of Ais are nonfunctioning, the rate of functioning adrenal adenomas is not uncommon. The risk of malignancy appears to be low in smaller masses. Additionally, in cases of obvious enlargement of adenoma size during the follow-up period, more attention should be paid to hormonal evaluation.

## Figures and Tables

**Table 1:** The baseline demographic characteristics and radiological features of 431 patients with adrenal incidentalomas.

Parameter	

Age (years) (mean ± SD)	55.4 ± 11.5

Sex, n (%)	
Female	301 (69.8%)
Male	130 (30.2%)

BMI (kg/m^2^) (mean ± SD)	29.3 ± 4.3

SBP (mmHg) (mean ± SD)	130.9 ± 13.8

DBP (mmHg) (mean ± SD)	81.9 ± 8.7

Comorbidity diseases	
None, n (%)	170 (39.4%)
Hypertension, n (%)	103 (23.9%)
Diabetes mellitus (n, %)	31 (7.2%)
Prediabetes, n (%)	16 (3.7%)
Hyperlipidemia (n, %)	27 (6.3%)
Coronary artery disease (n, %)	6 (1.4%)
Other (n, %)	78 (18.1%)

Tumor diameter (mm) (mean ± SD)	25.9 ± 14.3

Tumor size	
<40 mm, n (%)	378 (87.7%)
40–60 mm, n (%)	35 (8.1%)
>60 mm, n (%)	18 (4.2%)

Adenoma location	
Right, n (%)	165 (38.3%)
Left, n (%)	185 (42.9%)
Bilateral, n (%)	81 (18.8%)

Tumor density on CT with washout analysis	
≤10 HU, n (%)	294 (79.2%)
>10 HU with washout ≥50%, n (%)	32 (8.6%)
>10 HU with washout <50%, n (%)	45 (12.1%)

Shape irregularity, n (%)	10 (2.3%)

Heterogeneous appearance, n (%)	64 (14.8%)
Homogeneous appearance, n (%)	367 (85.2%)

Necrosis, n (%)	6 (1.4%)

Invasion, n (%)	3 (0.7%)

BMI: body mass index, SBP: systolic blood pressure, DBP: diastolic blood pressure, CT: computed tomography, SD: standard deviation, HU: Hounsfield unit.

**Table 2:** Comparison of the baseline characteristics of patients with nonfunctioning AIs and functioning AIs.

	Nonfunctioning AIs n = 330 (76.6%)	Functioning AIs n = 101 (23.4%)	p value

Age (years) (mean ± SD)	55.9 ± 11.1	53.8 ± 12.6	0.096
			
Female, n (%)	220 (66.7%)	81 (80.2%)	**0.009**
			
Male, n (%)	110 (33.3%)	20 (19.8%)	
			
BMI, (kg/m^2^) (mean ± SD)	29.4 ± 4.37	29.0 ± 3.96	0.348
			
Follow-up time, (years)	3.25 ± 2.05	3.05 ± 2.14	0.426
			
Baseline tumor size, (mm) (mean ± SD)	23.3 ± 13.0	29.9 ± 14.1	**<0.001**
			
Follow-up tumor size, (mm) (mean ± SD)	22.1 ± 10.4	29.6 ± 13.2	**<0.001**
			
Variation in mass size, (mm) (mean ± SD)	−0.50 ± 5.8	1.8 ± 5.4	**0.004**
			
Tumor size ≤40 mm, n (%)	305 (92.4%)	73 (72.3%)	**<0.001**
Tumor size 40–60 mm, n (%)	13 (3.9%)	22 (21.8%)	
Tumor size ≥60 mm, n (%)	12 (3.6%)	6 (5.9%)	
			
≤10 HU, n (%)	275 (83.3%)	53 (52.5%)	**<0.001**
>10 HU with washout ≥50%, n (%)	28 (8.5%)	9 (8.9%)	
>10 HU with washout <50%, n (%)	27 (8.2%)	39 (38.6%)	

AIs: adrenal incidentalomas, SD: standard deviation, BMI: body mass index, HU: Hounsfield unit.

**Table 3:** Comparison of the clinical and laboratory characteristics at baseline and follow-up patients with nonfunctioning Ais.

	Baseline	Follow-up	p value

Adrenal tumor size (mean ± SD)			
Right-sided	25.2 ± 12.0	25.5 ± 12.6	0.320
Left-sided	21.0 ± 9.4	20.2 ± 8.9	0.134
Bilateral	22.1 ± 8.5	22.8 ± 8.4	0.602

ACTH (mean ± SD) (pg/mL)	18.9 ± 16.08	19.4 ± 17.7	0.847

Midnight salivary cortisol (mean ± SD) (μg/dL)	2.1 ± 1.4	2.4 ± 0.5	0.598

24-h urinary free cortisol (mean ± SD) (μg/24 h)	82.0 ± 48.5	54.4 ± 35.1	0.067

Plasma cortisol after DST (mean ± SD) (μg/dL)	1.6 ± 1.9	1.5 ± 1.3	0.505

DHEA-S (mean ± SD) (μg/dL)	108.7 ± 123.5	77.8 ± 68.7	0.065

17-Hydroxyiprogesterone (mean ± SD) (ng/dL)	13.4 ± 21.3	4.6 ± 5.8	0.428

Aldosterone (mean ± SD) (ng/dL)	22.3 ± 12.1	22.6 ± 13.9	0.866

Plasma renin activity (mean ± SD) (ng/mL/h)	3.4 ± 3.3	3.9 ± 4.2	0.349

Fasting blood glucose (mean ± SD) mg/dL	102.8 ± 29.6	109.4 ± 36.4	**0.002**

Fasting insulin (mean ± SD) uIU/mL	8.8 ± 2.7	14.2 ± 7.7	**< 0.001**

HOMA-IR	2.1 ± 0.9	3.1 ± 1.9	**0.004**

Total cholesterol (mean ± SD) mg/dL	209.9 ± 43.6	214.1 ± 44.2	0.229

HDL-C (mean ± SD) mg/dL	49.1 ± 12.2	50.9 ± 13.1	0.005

LDL-C (mean ± SD) mg/dL	131.4 ± 35.9	129.1 ± 38.5	0.354

Triglyceride, (mean ± SD) mg/dL	148.9 ± 77.3	155.4 ± 79.4	0.249

DST: dexamethasone suppression test, ACTH: adrenocorticotropin hormone, SD: SD: standard deviation, DHEA-S: dehydroepiandrosterone sulfate.
